# Bibliometric study of immunotherapy for hepatocellular carcinoma

**DOI:** 10.3389/fimmu.2023.1210802

**Published:** 2023-08-04

**Authors:** Zhiyi Li, Ying Zhang, Baipan Zhang, Rui Guo, Minhua He, Zi-Ling Liu, Lei Yang, Hong Wang

**Affiliations:** ^1^ Cancer Center, The First Hospital of Jilin University, Changchun, China; ^2^ Department of Clinical Laboratory, First Affiliated Hospital of Jilin University, Changchun, China; ^3^ College of Computer Science and Technology, Jilin University, Changchun, China

**Keywords:** hepatocellular carcinoma, immunotherapy, bibliometric, Citespace, VOSviewer, HCC, liver cancer

## Abstract

**Background:**

Hepatocellular carcinoma (HCC), recognized as a significant global health concern, ranks as the sixth most prevalent form of cancer and is the third leading cause of cancer-associated mortality. Over half of HCC patients are diagnosed at advanced stages, an unfortunate phenomenon primarily attributed to the liver’s robust compensatory mechanisms. Given the limited availability of donor livers, existing clinical surgical approaches have yet to provide universally applicable treatment strategies offering substantial prognostic improvement for late-stage cancer. Although the past few decades have witnessed significant advancements in chemotherapy and targeted therapy for HCC, the emergence of drug resistance poses a substantial impediment to their successful execution. Furthermore, issues such as diminished quality of life post-treatment and high treatment costs warrant critical attention. Consequently, the imperative for an effective treatment strategy for advanced liver cancer is unequivocal. In recent years, notable progress in the development and application of immunotherapy has sparked a revolution in advanced liver cancer treatment. This study aims to elucidate a more comprehensive understanding of the current landscape, knowledge framework, research focal points, and nascent breakthrough trends in the domain of immunotherapy for hepatocellular carcinoma via bibliometric analysis.

**Method:**

Our study involved conducting a comprehensive literature search spanning from 1999 through December 31, 2022, by utilizing the Science Citation Index Expanded (SCI-Expanded) database. Our aim was to amass all the papers and reviews related to immunotherapy for hepatocellular carcinoma. Our search strategy yielded a total of 4,486 papers. After exclusion of self-citations, we focused our analysis on 68,925 references. These references were cited 119,523 times (excluding 97,941 self-citations), boasting an average citation frequency of 26.64 times per paper, and achieved an h-index of 135. We employed analytical software tools like Citespace and VOSviewer to perform an intricate analysis of the amassed literature, covering various aspects, including geographical location, research institutions, publishing journals, authors, references, and keywords. Our method incorporated timeline analysis, burst detection, and co-occurrence analysis. The application of these tools facilitated a thorough evaluation of research hotspots, knowledge structure, and emerging advancements within the sphere of immunotherapy for hepatocellular carcinoma.

**Results:**

Our bibliometric analysis disclosed a noteworthy escalation in the number of publications in the realm of hepatocellular carcinoma immunotherapy during the years 2021-2022, surpassing the aggregate number of papers published in the preceding decade (2011–2020). This surge underscores a sharp upturn in research interest within this field. Additionally, the research hotspot in hepatocellular carcinoma immunotherapy has perceptibly deviated from the preceding decade’s trends. In terms of geographical distribution, China emerged as the leading country, producing 50.08% of the total publications. This was followed by the United States, with 963 papers, and Japan, contributing 335 papers. Among research institutions, Sun Yat-sen University was the most prolific, while Tim F. Greten stood out as the most published author with 42 papers to his credit. A co-reference network examination uncovered a shift in research emphasis within the field of hepatocellular carcinoma immunotherapy, highlighting the evolving nature of this important area of study

**Conclusion:**

Our bibliometric study highlights the significant evolution and growth in HCC immunotherapy research over the past two decades. Looking ahead, research will focus on improving the microenvironment post-drug resistance from immune combination therapy, harnessing adoptive cellular immunity (as CAR-T), subclassify the population and developing new tumor markers. Incorporation of technologies such as nanotechnology, microbiology, and gene editing will further advance HCC treatments. This progressive trajectory in the field promises a brighter future for individuals suffering from HCC.

## 1 Introduction

Primary liver cancer manifests in several forms, inclusive of hepatocellular carcinoma (HCC) (75%-85%), intrahepatic cholangiocarcinoma (10%-15%), and other variants. HCC constitutes a malignant epithelial tumor, primarily originating from hepatic cells, with chief risk factors being chronic infections with Hepatitis B virus (HBV) or Hepatitis C virus (HCV). Secondary risk factors entail exposure to aflatoxin, tobacco smoking, alcohol consumption, obesity, and diabetes. HCC ranks as the sixth most prevalent cancer globally (1), and it is the second leading cause of cancer-related mortality (2).

While the advent of a vaccine against the hepatitis virus might marginally ameliorate this predicament in the future, escalating trends of obesity, metabolic syndrome, and diabetes presage a rise in the incidence of HCC (2). It is projected that by 2025, liver cancer cases will exceed one million per annum (3).

Given the liver’s substantial compensatory capacity, a substantial proportion of patients are diagnosed with advanced-stage HCC (1). The scarcity of donor livers coupled with the dearth of universally applicable clinical surgical techniques that offer a favorable prognosis for late-stage cancer amplify the complexity of this issue. Despite chemotherapy and targeted therapy for HCC achieving considerable strides over the past decades, the emergence of drug resistance has evolved into a significant impediment to their successful deployment. Furthermore, the conundrums of diminished post-treatment survival quality and escalating costs demand urgent attention. Hence, the development of an effective strategy for advanced liver cancer is a pressing necessity.

Immunotherapy has evolved as an innovative, effective, and promising therapeutic intervention for liver cancer, especially for cases of advanced, chemoresistant hepatocellular carcinoma (HCC). A multitude of immunotherapeutic strategies have been developed to address HCC, which include activated lymphocyte therapy, natural killer cell therapy, dendritic cell therapy, tumor infiltrating lymphocyte therapy, gene-modified T cell therapy, CAR T cell therapy, TCR engineered T cell therapy, among others. Gene-modified T cell therapies such as CAR T or TCR-T cell therapy have garnered notable recognition as groundbreaking therapeutic strategies for HCC (4).

Immunotherapy has surfaced as an emergent therapeutic modality for HCC (5), demonstrating substantial efficacy and potential, thereby becoming an indispensable component of the treatment regimen for chemoresistant HCC. Bibliometrics, which comprises the quantitative analysis of knowledge carriers, typically literature, within a specific field, harnesses mathematical and statistical methods. The objective of this approach is to assess research hotspots, determine the architecture of domain knowledge, and predict potential breakthroughs (5, 6). In this regard, analytical tools like CiteSpace (7), VOSviewer (8), and HistCite (9) are frequently employed due to their capacity to analyze and visualize vast quantities of literature data (10–13).

A prior bibliometric review of immunotherapy for HCC spanning from 2011 to 2020 demonstrated an intriguing trend: the number of publications in 2021 and 2022 (1,864) surpassed the total number of publications in the previous decade, from 2011 to 2020 (1,641). This observation holds true whether analyzed from a macro perspective (a 114% increase in the sample size) or from a micro level that explores frontier literature information. Evidently, the focus and trajectory of research in HCC immunotherapy have undergone significant shifts in recent years. Thus, it becomes essential to re-evaluate and analyze the contemporary state and emerging hotspots in this field.

## 2 Materials and methods

### 2.1 Scientometric analysis

**Figure 1:**
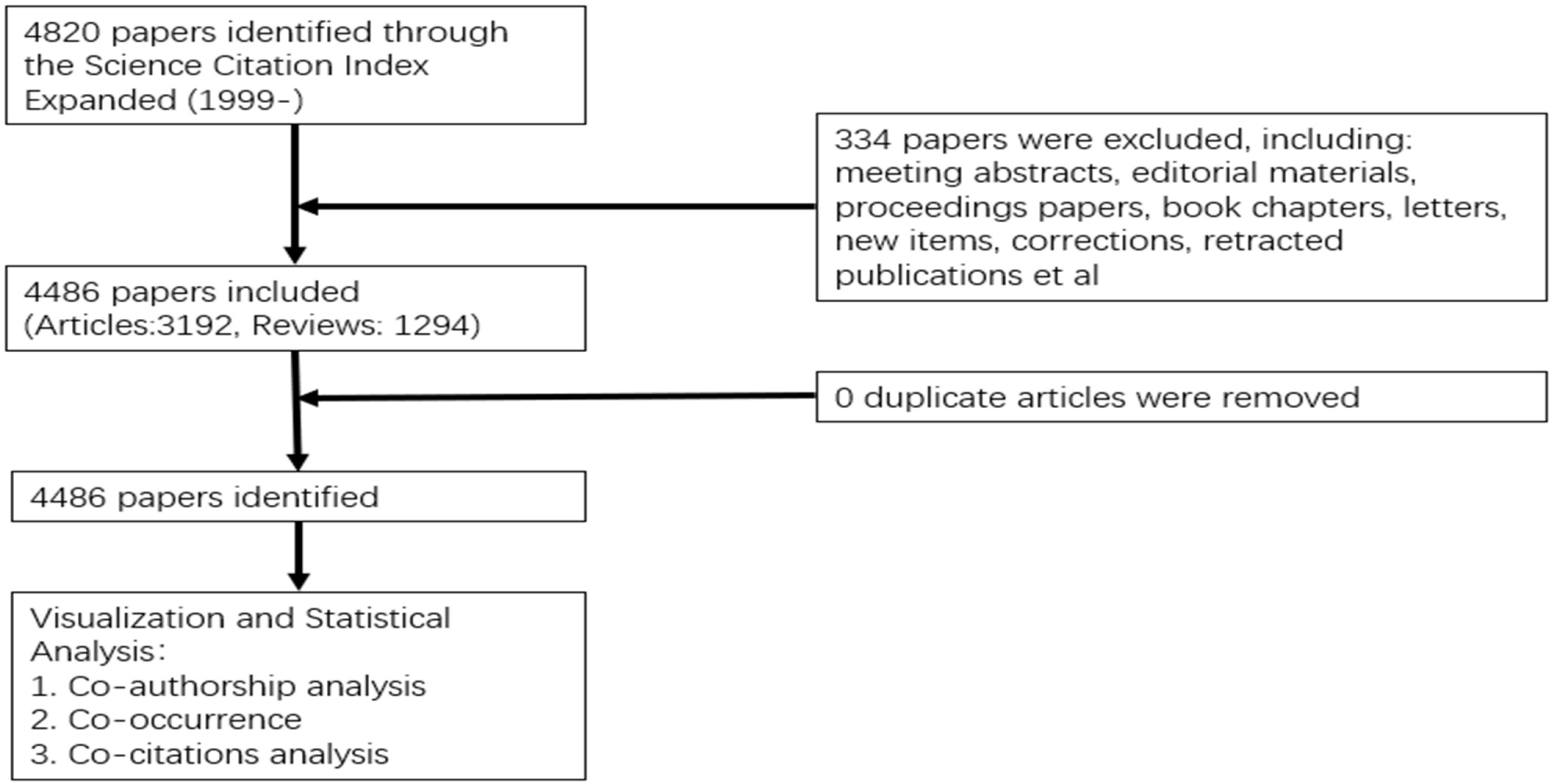
Flowchart of the literature searching and screening in the study.

We utilized the Web of Science Core Collection (WoSCC) to procure articles in the domain of immunotherapy from 1999 through 2022, drawing data from the Science Citation Index Expanded (SCI-Expanded). Our search strategy was informed by terms derived from the ClinicalTrials.gov database, utilizing TS= (liver cancer) AND TS=(immunotherapy). These search terms were subsequently revised and refined to optimize the search logic. Our primary search terms included TS1=“liver cancer” OR “Liver Neoplasm” OR “liver tumors” OR “Hepatocellular Cancers” OR “Hepatoma” OR “Liver Carcinoma” OR “Hepatic tumor” OR “Hepatic cancer” OR “Cancer of Liver” OR “cancer of the liver” OR “hepatic neoplasms” OR “Neoplasm of liver” OR “liver and intrahepatic bile duct carcinoma” OR “liver and intrahepatic biliary tract cancer” OR “Tumor of liver” OR “liver malignant tumors” OR “Neoplasm of the liver” OR “Hepatocellular Carcinoma”, and TS2=“Immunotherapeutic” OR Immunotherapy OR Immunotherapies OR immunotherapeutics. Detailed information regarding the literature search is presented in the **Supplementary Material**. We restricted our search to papers and reviews, and confined the language to English (**Figure 1**).

To mitigate any potential bias introduced by database updates, the search was executed on January 29, 2023, yielding a total of 4,486 full records from the Web of Science (WoS) database. These full records were downloaded as both Plain Text Files and Tab Delimited Files.

The WoS platform offers an annual distribution of publications and citations. Notably, the Web of Science Core Collection (WoSCC) serves as the fundamental data source for Citespace (6.1.R3) and VOSviewer (1.6.19).

#### 2.1.1 Citespace (6.1.R3)

The initial search procured 4,486 results, which were downloaded as plain text files. Upon inspection and removal of duplicate entries, we confirmed the absence of duplicates. Subsequent statistical analysis revealed participation from 3,896 institutions across 79 countries and contributions from 23,574 authors. The papers were disseminated across 848 distinct journals and employed a total of 11,816 keywords, in addition to 6,386 author-specific keywords.

Prior to conducting the analysis, Citespace was configured with the following parameters: 1) Time slice: January 1999-December 2022; 2) Pruning: Pathfinder/pruning sliced network/pruning the merged network; 3) Other functions: Retained at default settings. The examination of countries, institutions, citation references, and keywords associated with the study (inclusive of timeline analysis, burst analysis, and co-occurrence analysis) was executed using Citespace.

#### 2.1.2 VOSviewer(1.6.19)

Download 4486 search results from WoSCC in Tab Delimited File format. Use VOS viewer to analyze countries, institutions, authors, research hotspots, and more in the field.

## 3 Results

### 3.1 Scientometric analysis

#### 3.1.1 Distribution of publications and citations by year

Our study yielded a total of 4,486 documents via the aforementioned search strategy. These documents accumulated 72,491 citations, excluding 68,925 self-citations, and were cited 119,523 times, excluding 97,941 self-citations. The average citation frequency was 26.64 per paper. The h-index was established at 135, denoting that 135 references were cited more than 135 times, which underlines their significance and widespread acknowledgment in the field.

##### 3.1.1.1 Temporal trends in publication and citation distribution

**Figure 2:**
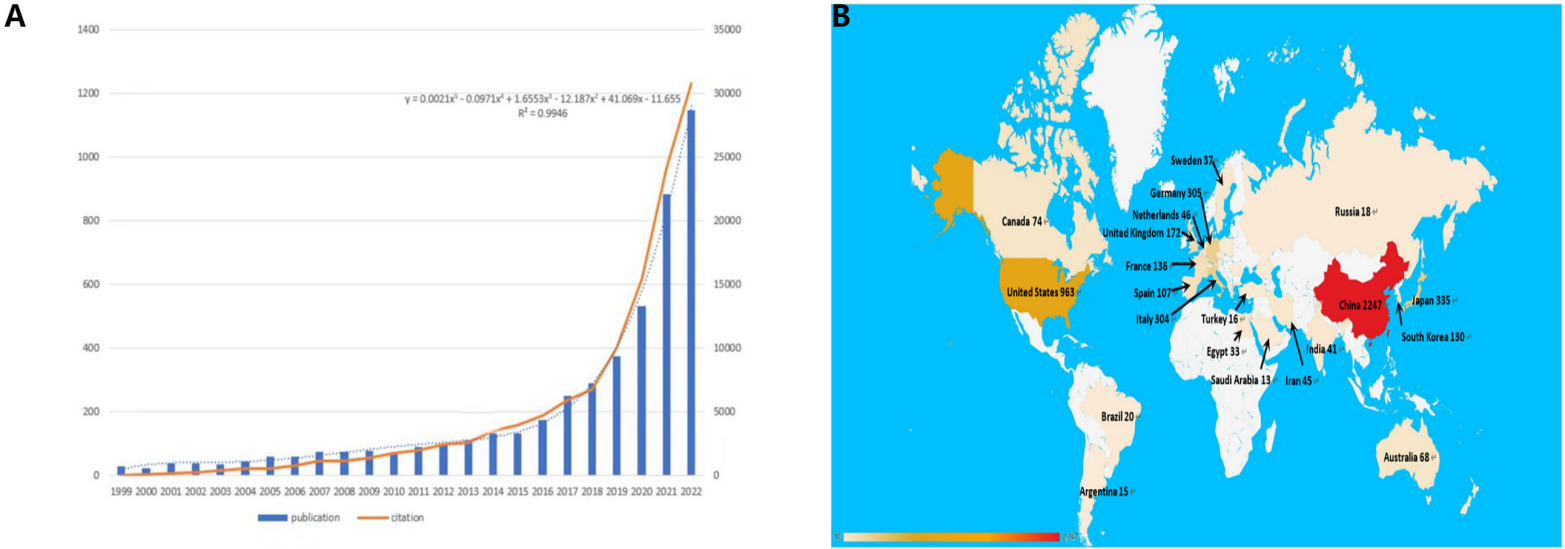
**(A)** The polynomial curve fitting of publication and citation growth in immunotherapy of HCC. **(B)** Analysis of countries engaged in immunotherapy research in relation to HCC: the top most productive countries/regions.

**Figure 2A** presents the distribution of publications and citations, highlighting a consistent upward trajectory from 1999 through 2022. Microsoft Excel LTSC MSO was employed to process the data and establish a polynomial regression model. The escalating number of publications and citations in the field of interest over time implies a surge in interest and research activity in this area.

##### 3.1.1.2 Geographical disparity in publication volume

**Figure 2B** delineates the distribution of publications by country. Discrepancies in the volume of publications across countries could be attributable to factors such as economic considerations, healthcare investments, dietary habits, and prevalence of HBV infection. These factors warrant more detailed exploration later in this study.

#### 3.1.2 Related countries (or regions) and institutions of this field

##### 3.1.2.1 Country analysis

**Figure 3:**
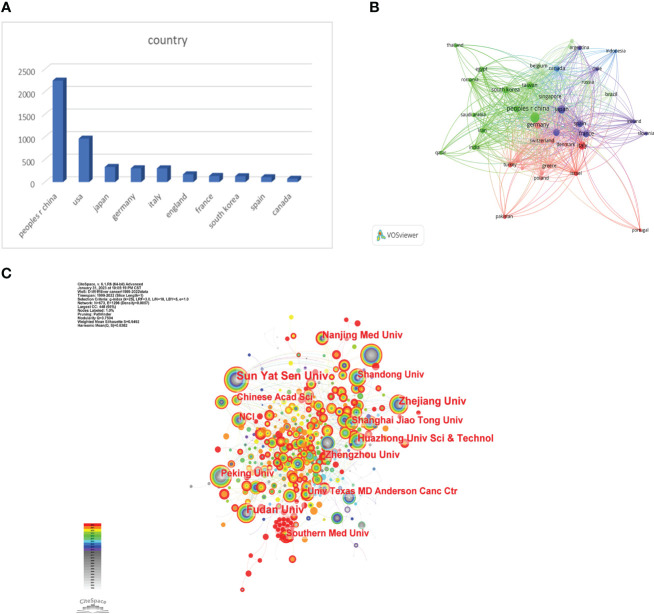
**(A)** The distributions of countries in terms of publications. **(B)** A network map showing countries publishing more than 10 papers in this field involved in the research on immunotherapy in relation to HCC. **(C)** A network map showing institutions involved in immunotherapy research in relation to HCC.

We used VOSviewer to analyze the countries and regions that have published papers in the field of immunotherapy for hepatocellular carcinoma. The analysis revealed that 32 countries or regions have published more than ten papers in this area (**Figure 3B**). China took the lead in publishing the most articles, with a total of 2,247 articles, accounting for 50.08% of the total publications. It was followed by the United States with 963 articles (21.46%) and Japan with 335 articles (7.46%) (**Figure 3A**).

##### 3.1.2.2 Institution analysis

Research institutions were examined utilizing Citespace. The analysis drew upon contributions from a total of 3,896 institutions. Sun Yat-sen University held the top rank with the highest number of published papers (186) (**Figure 3C**). All top 10 publishing institutions hailed from China (**Table 1**). These findings offer valuable insights into the predominant countries and research institutions, along with their contributions to the field of immunotherapy for hepatocellular carcinoma. The preponderance of publications from China underscores its significant presence and contributions in this research arena. Sun Yat-Sen University is recognized as a key institution in terms of the volume of published papers.

**Table 1:** The top 10 institutions involved in research on immunotherapy in relation to HCC.

institution	count
sun yat sen univ	186
fudan univ	145
zhejiang univ	133
huazhong univ sci & technol	94
nanjing med univ	87
zhengzhou univ	87
shanghai jiao tong univ	85
peking univ	75
chinese acad sci	71
southern med univ	71

#### 3.1.3 Author

##### 3.1.3.1 Most prolific authors

**Figure 4:**
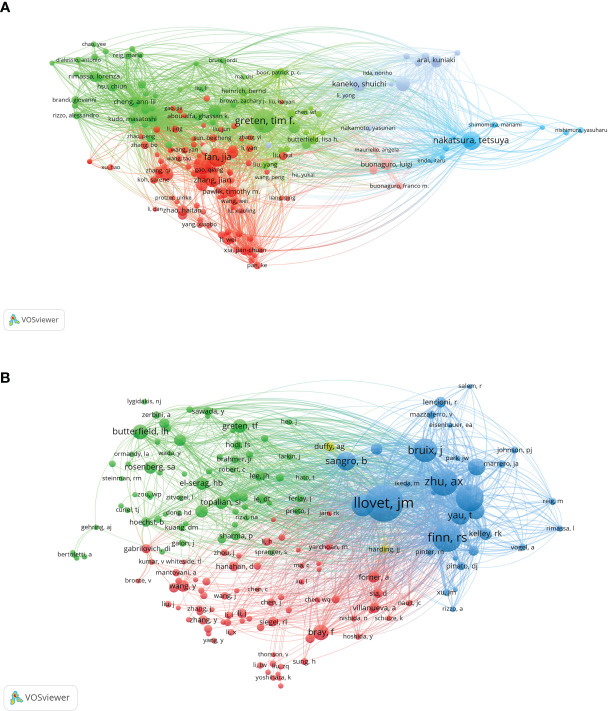
A network map showing authors. **(A)** highly published authors. **(B)** highly cited authors.

VOSviewer analysis revealed that over 20,000 researchers have contributed to studies focusing on immunotherapy for hepatocellular carcinoma (HCC). Among them, 107 prolific authors have published more than 10 papers on this subject. The author with the most publications is Greten, Tim F, with 43 papers. Fan, Jia and Nakatsura, Tetsuya follow closely, each with 33 publications, and Zhou, Jian with 31 papers (**Figure 4A**). In terms of citations, Finn, Richard S had the most, with a citation volume of 4502, followed by Llovet, Josep M with 3526 citations (**Table 2**). Notably, the two most frequently cited papers were both authored by Finn, Richard S and Llovet, Josep M.

**Table 2:** The top 10 published authors and co-cited authors involved in research on immunotherapy in relation to HCC.

rank	author	count	co-cited author	count
1	greten, tim f.	43	llovet, jm	2199
2	fan, jia	33	finn, rs	1444
3	nakatsura, tetsuya	33	zhu, ax	1357
4	zhou, jian	31	kudo, m	1354
5	kaneko, shuichi	25	bruix, j	1069
6	zhang, jian	25	el-khoueiry, ab	957
7	mizukoshi, eishiro	24	sangro, b	761
8	bertoletti, antonio	22	yau, t	749
9	sangro, bruno	21	abou-alfa, gk	660
10	wang, yu	20	cheng, al	642

##### 3.1.3.2 Co-citation analysis

A co-citation analysis using VOSviewer was conducted to illustrate the functional and thematic influence of authors who have been cited more than 100 times in the field of HCC immunotherapy (**Figure 4B**). The analysis generated a network of interconnected authors, consisting of 175 authors. Each node in the graph represents an author. The size of the circle correlates positively with the number of articles published by that author, with the transparency increasing with fewer articles. The number and thickness of the lines connecting the circles denote the co-occurrence relationship between authors. Authors denoted by the same color belong to the same cluster, indicating that their work is frequently cited together. The results of the author analysis and co-cited author analysis showed a close correlation. Highly productive authors tend to appear together more than others.

According to the analysis, among the top ten cited authors, Llovet, JM ranks first with 2199 citations, followed by Finn, RS with 1444, and Zhu, AX with 1357 citations. In addition, several active co-citation relationships are noticeable, such as between Llovet, JM and Finn, RS, and Zhu, AX and Bruix, J. This symbiotic analysis provides insights into the collaborative relationships and thematic influence among these authors, further enriching our understanding of the research landscape in this area.

#### 3.1.4 Journals

##### 3.1.4.1 Most prolific journals and citation relationships

**Figure 5:**
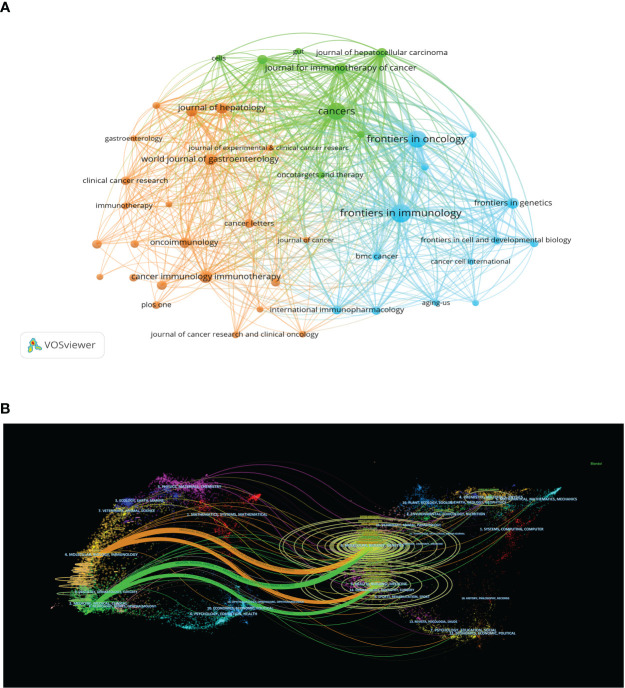
**(A)** Co-citation analysis of the journals. **(B)** Overlay graph of journal themes.

A total of 848 academic journals have published articles related to liver cancer immunotherapy. The journal with the most publications in this area is “Frontiers in Immunology”, with 183 articles and an impact factor of 7.3 for 2022. Among the journals that have published more than 50 articles, the “Journal of Hepatology” ranks highest in impact factor (25.7 in 2020) with 66 articles, followed by “Hepatology” with an impact factor of 13.5 in 2020 and 59 articles (**Table 3**). As shown in **Figure 5A**, there are positive citation relationships among different journals.

**Table 3:** The top 10 published journals involved in research on immunotherapy in relation to HCC.

rank	journal	count	percent	IF(2022)#
1	frontiers in immunology	183	4.07%	7.3
2	cancers	159	3.54%	5.2
3	frontiers in oncology	158	3.52%	4.7
4	cancer Immunology Immunotherapy	78	1.73%	5.8
5	world journal of gastroenterology	74	1.64%	4.3
6	journal for immunotherapy of cance	67	1.49%	10.9
7	journal of hepatology	66	1.47%	25.7
8	frontiers in genetics	65	1.44%	3.7
9	hepatology	59	1.31%	13.5
10	oncoimmunology	58	1.29%	7.2

##### 3.1.4.2 Dual map overlay analysis

**Figure 5B** provides a dual map overlay of journal themes. The journals that cite articles are located on the left side of the map, while the journals that are cited are on the right side. The labels close to the emitting region represent the respective disciplines, with each label centered around the cluster centroid of the corresponding journals. Colored lines, moving from left to right, depict the citation pathways. The longer the vertical axis of the ellipse, the more papers the journal publishes, and the longer the horizontal axis, the more authors it has. There are four major citation pathways. Two orange pathways suggest that journals from the molecular/biology/genetics and health/nursing/medicine domains are frequently cited in molecular/biology/immunology journals. The two green paths indicate that research journals from the molecular/biology/genetics and health/nursing/medicine domains are frequently cited in medicine/medical/clinical journals

#### 3.1.5 Citation

##### 3.1.5.1 Top 10 most cited articles

The table below (**Table 4**) lists the top 10 most cited articles in this field. Among them, the study titled “Nivolumab in patients with advanced hepatocellular carcinoma (CheckMate 040): an open-label, non-comparative, phase 1/2 dose escalation and expansion trial” by Anthony B El-Khoueiry et al. has been cited the most frequently (n=851). The prominence of this study within the field of immunotherapy for hepatocellular carcinoma suggests that its findings and conclusions have made a substantial impact, influencing subsequent research and discussions.

**Table 4:** The top 10 literatures involved in immunotherapy research in relation to HCC.

reference	count	doi
el-khoueiry ab, 2017, lancet, v389, p2492,	851	10.1016/s0140-6736 (17)31046-2
llovet jm, 2008, new engl j med, v359, p378	684	10.1056/nejmoa0708857
finn rs, 2020, new engl j med, v382, p1894	567	10.1056/nejmoa1915745
bray f, 2018, ca-cancer j clin, v68, p394	554	10.3322/caac.21492
zhu ax, 2018, lancet oncol, v19, p940	539	10.1016/s1470-2045 (18)30351-6
kudo m, 2018, lancet, v391, p1163	455	10.1016/s0140-6736 (18)30207-1
bruix j, 2017, lancet, v389, p56	412	10.1016/s0140-6736 (16)32453-9
finn rs, 2020, j clin oncol, v38, p193	340	10.1200/jco.19.01307
sangro b, 2013, j hepatol, v59, p81	338	10.1016/j.jhep.2013.02.022
abou-alfa gk, 2018, new engl j med, v379, p54	283	10.1056/nejmoa1717002

##### 3.1.5.2 Co-citation analysis

**Figure 6:**
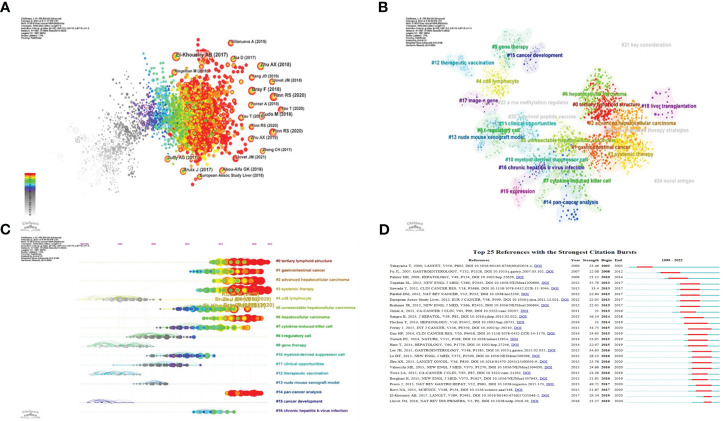
Application of CiteSpace. **(A)** Co-citation analysis of the literature. **(B)** Cluster analysis of co-cited literature network. **(C)** The time axis view of co-citation literature analysis data. **(D)** High reference outbreak analysis of clustering data.

We utilized CiteSpace to conduct a co-citation analysis of the literature, aiming to uncover the progression of immunotherapy research in hepatocellular carcinoma. The findings are illustrated in the figure above (**Figure 6A**). Most of the highly cited literature emerged in recent years, indicating a rapid advancement and a vast array of results within this research area. For a more nuanced analysis, we selected the top 10 articles with the highest co-citation count.

##### 3.1.5.3 Cluster analysis

The network of co-cited literature generated by our analysis yielded several clusters, which are represented in **Figure 6B**. The figure displays 25 clusters, with the first labeled “# 0 tertiary lymphoid structure,” the second “# 1 gastrointestinal cancer,” and the third “# 2 advanced hepatocellular carcinoma.”

##### 3.1.5.4 Timeline view of co-citation data

The co-citation literature analysis data, processed for a timeline view, exhibits nodes symbolizing individual references; larger nodes denote a higher reference count. Nodes on the left represent earlier references, while those on the right represent more recent ones. Nodes positioned along the same line represent a cluster, identified by the label # on the right side.

The timeline view reveals several relatively new research hotspots, including “#0 tertiary lymphoid structure,” “#1 gastrointestinal cancer,” “#2 advanced hepatocellular carcinoma,” “#3 system therapy,” “#5 unresectable hepatocellular carcinoma,” “#6 hepatocellular carcinoma,” and “#14 pan-cancer analysis” (**Figure 6C**).

##### 3.1.5.5 Citation burst analysis

We performed a citation burst analysis of the cluster data using CiteSpace, a method which highlights the degree of citation for specific documents over time. The green line represents the period from 1999 to 2022, while the red line indicates the duration of each citation burst (**Figure 6D**).

These figures offer insights into the temporal evolution of research topics, spotlighting pivotal clusters and citation bursts within the field of immunotherapy for hepatocellular carcinoma. They underscore the dynamic nature of the research landscape and the emergence of novel focal points over time.

#### 3.1.6 Analysis of keyword co-occurrence

**Figure 7:**
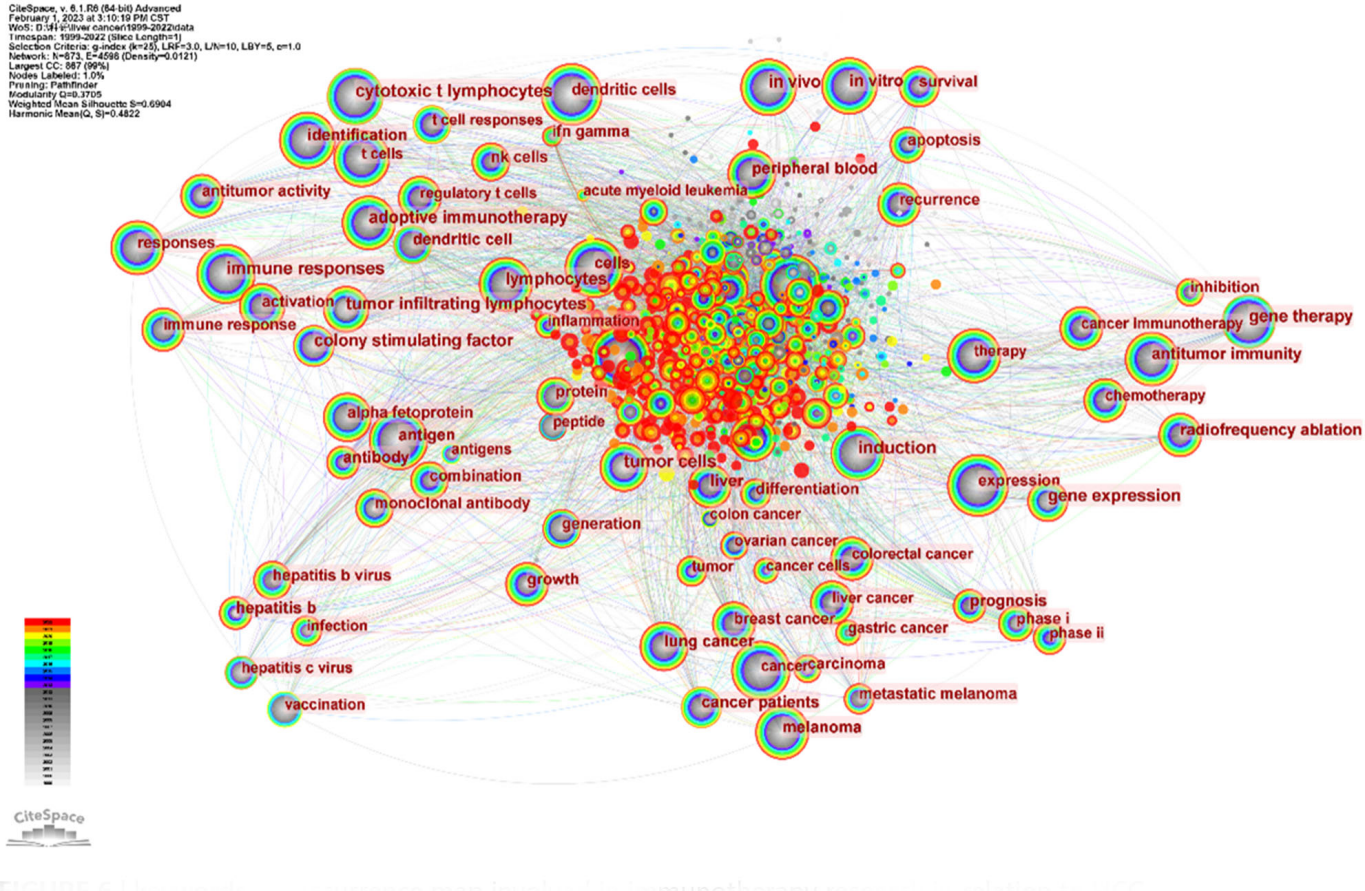
keyword co-occurrence map involved in immunotherapy research in relation to HCC.

Keyword analysis, made possible by CiteSpace, allows us to generate a co-occurrence map and visualize the relationship between keywords. This strategy efficiently reflects the core framework and research hotspots of a certain field, and to a certain extent, indicates new research breakthroughs (**Figure 7**). The keywords identified in our study are classified into four categories: liver virus, liver tumor, tumor immune response, and immunotherapy. A list of the most frequently occurring keywords can be found in **Table 5**.

**Table 5:** The top ten keywords appeared the most.

rank	keyword	count
1	immunotherapy	2181
2	hepatocellular carcinoma	1594
3	hepatocellular-carcinoma	1176
4	cancer	1047
5	expression	728
6	sorafenib	503
7	dendritic cells	485
8	therapy	450
9	t-cells	403
10	prognosis	391

Through the co-occurrence of these keywords, we can gain insights into the primary themes, connections, and emerging research trends in the field of immunotherapy for hepatocellular carcinoma. The emphasis on the interplay between liver viruses, liver tumors, tumor immune responses, and immunotherapy underscores the intricate dynamics of these factors in the development and treatment of hepatocellular carcinoma.

In conclusion, these findings illuminate the fundamental structure of the field. It is clear that immunotherapy for hepatocellular carcinoma is often associated with secondary complications arising from hepatitis virus infection. This underscores the importance of developing comprehensive strategies that address both the primary disease and its related complications.

#### 3.1.7 Keyword timeline view

Cluster analysis of keywords is indeed a powerful tool for identifying major research themes within a specific field. Through the use of CiteSpace in this study, a cluster analysis was conducted on the keywords associated with immunotherapy for hepatocellular carcinoma. The number of clusters was determined based on the size of each cluster, with the largest cluster being labelled as #0.

**Figure 8:**
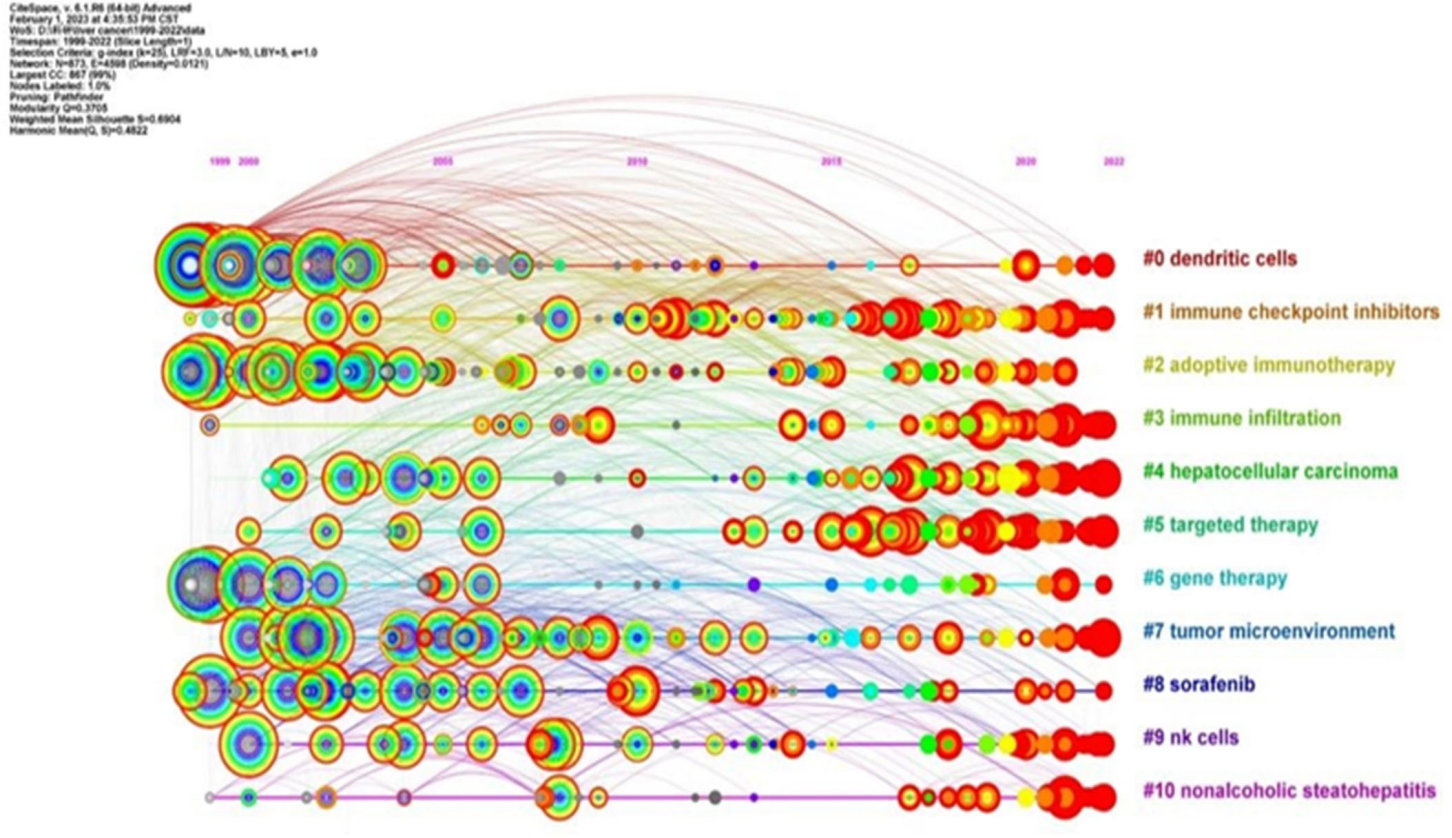
Cluster analysis of keywords involved in immunotherapy research in relation to HCC.

This analysis resulted in 11 clusters, which were subsequently analyzed using a timeline view in CiteSpace. These clusters included #0 dendritic cells, #1 immune checkpoint inhibitors, #2 adoptive immunotherapy, #3 immune infiltration, #4 hepatocellular carcinoma, #5 targeted therapy, #6 gene therapy, #7 tumor microenvironment, #8 sorafenib, #9 NK cells, and #10 nonalcoholic steatohepatitis. The timeline view generated from this cluster analysis is illustrated in **Figure 8**.

The representation of the research field (immunotherapy for HCC) from the perspective of hotspots and development status, is divided into the same four categories identified in the co-occurrence analysis: 1) Hepatitis (#10), 2) Liver tumors (#4, #7), 3) Tumor immune response (#0, #1, #3, #9), and 4) Immunotherapy (#2, #5, #6, #8) (**Figure 8**). Moreover, the analysis revealed that the interest in clusters #3, #4, and #5 has consistently risen and remained high in recent years. This suggests that immunity and immunotherapy have consistently attracted significant attention in the field of hepatocellular carcinoma. This trend is likely to continue as researchers strive to enhance therapeutic strategies and patient outcomes.

In conclusion, the cluster analysis and timeline view provide valuable insights into research trends, thematic progression, and the evolving importance of specific keywords within the realm of hepatocellular carcinoma immunotherapy.

#### 3.1.8 Analysis of keyword burst

Burst keywords analysis using CiteSpace indeed helps to identify the most rapidly emerging keywords, also known as burst keywords. These are considered to have generated widespread attention within the academic community, serving as key indicators of research trends in the field. Through burst keywords analysis, the 25 most representative keywords were selected from a pool of 11,816 keywords to identify the hotspots of research. These keywords were sorted by duration, start time, and burst intensity.

(The green line illustrates the period from 1999 to 2022, and the duration of each burst keyword is represented by the red line.)

##### 3.1.8.1 Earliest burst keyword

**Figure 9:**
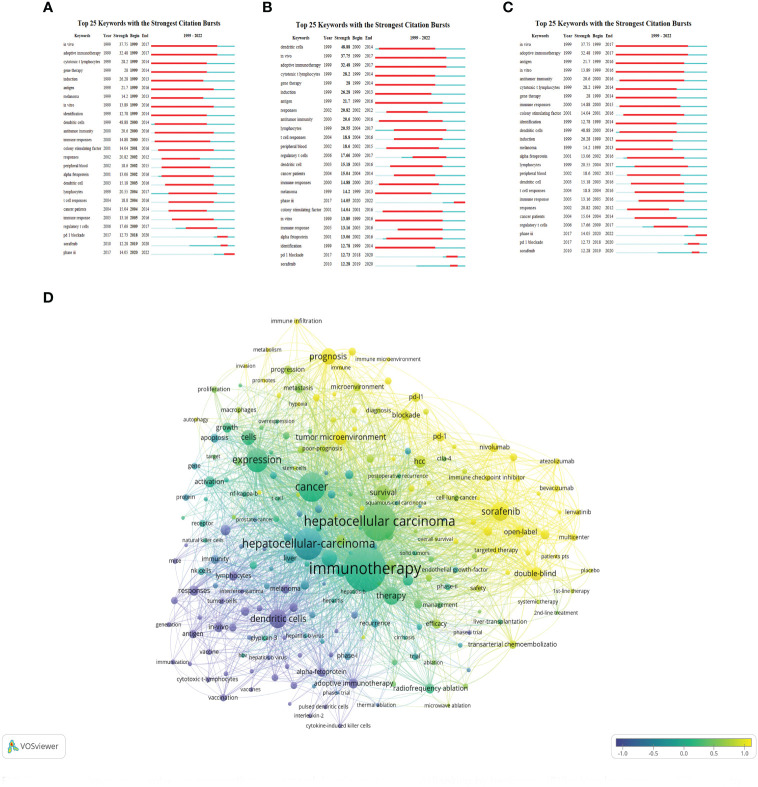
Burst keywords involved immunotherapy research in relation to HCC. **(A)** Ranking by beginning. **(B)** Ranking by strengths. **(C)** Ranking by durations. **(D)** keywords are clustered on the time scale and output as map.

“*In vivo*” emerged as the earliest burst keyword in 1999 (**Figure 9A**), implying that the concept and application of *in vivo* research methods attracted significant interest in the field of HCC immunotherapy during that period.

##### 3.1.8.2 Strongest burst keyword

“Dendritic cells” displayed the highest burst intensity (**Figure 9B**), indicating significant attention and research activity around this keyword. Dendritic cells are a type of immune cell known for their vital role in modulating immune responses.

##### 3.1.8.3 Longest-lasting burst keyword

“*In vivo*” also demonstrated the longest duration of burst activity (**Figure 9C**), reflecting a sustained interest in *in vivo* research methodologies over the analyzed period.

##### 3.1.8.4 Categorization of burst keyword

The analysis identified 25 burst keywords which can be broadly categorized into two groups: 1) Body immunity associated with HCC, and 2) Immunotherapy techniques for HCC. The burst keywords were more concentrated from 1999 to 2017, and in combination with the triangular pattern presented by the keyword time map (created using VOSviewer to cluster keywords over a timeline), it suggests that HCC immunotherapy research is moving towards more specialized fields (**Figure 9D**).

Recent notable burst keywords include “phase iii” (14.05), “pd-1 blockade” (12.73), “sorafenib” (12.28), “dendritic cells” (48.88), “adoptive immunotherapy” (32.48), and “gene therapy” (14) - the numbers in parentheses represent the burst strength of each keyword. These keywords underline the focus on advanced clinical trials (phase iii), immune checkpoint inhibition (PD-1 blockade), and specific treatment modalities like sorafenib. Additionally, the significance of dendritic cells, adoptive immunotherapy, and gene therapy accentuates the importance of immunotherapeutic strategies in HCC research.

This analysis implies that the field of immunotherapy for hepatocellular carcinoma has seen considerable development and refinement over the past two decades, with more specific and targeted research areas becoming prominent. The concentration of burst keywords in the earlier years may indicate a period of rapid advancement and discovery in the field, while the recent burst keywords suggest a concentration on specific treatments and strategies for HCC immunotherapy. In essence, the burst keyword analysis provides valuable insights into the evolution and current status of research in the field of HCC immunotherapy.

## 4 Discussion

### 4.1 Immunotherapy for hepatocellular carcinoma

Immunotherapy has emerged as a promising frontier in the treatment landscape of hepatocellular carcinoma (HCC). This novel approach, which leverages the body’s immune system to tackle cancer, offers potential advantages over traditional treatment modalities such as surgery, chemotherapy, and radiotherapy. These conventional methods often yield limited efficacy, are frequently associated with significant side effects, and may contribute to multidrug resistance. However, immunotherapy provides a more targeted, less toxic therapeutic alternative, demonstrating considerable promise particularly in advanced HCC stages where conventional treatments may prove ineffective.

#### 4.1.1 Mechanisms of cancer immunotherapy

At its core, cancer immunotherapy strives to activate and amplify the immune system’s capabilities to detect, engage, and eliminate cancer cells. The primary therapeutic strategies in this realm include immune checkpoint inhibitors, adoptive cell therapy, therapeutic cancer vaccines, combined targeted therapy, and regional therapy combination.

#### 4.1.2 Immune checkpoint inhibitors

Immune checkpoint inhibitors function by addressing the overexpression of immune checkpoint molecules such as CTLA4, PD-1, and PD-L1 in the tumor microenvironment. This overexpression enables liver cancer cells to evade immune response (15). When these molecules bind with their respective receptors on immune cells, they inhibit their function, leading to suppression of anti-tumor immune responses. By targeting these molecules, inhibitors such as anti-CTLA4, anti-PD-1, and anti-PD-L1 antibodies demonstrate potential in liver cancer immunotherapy, as they block immunosuppressive signals and reactivate anti-tumor immune responses.

#### 4.1.3 Adoptive cell therapy

Adoptive cell therapy is a form of passive therapy that leverages effector cells like NK cells, lymphokine-activated killer cells (LAK), and cytokine-induced killer cells (CIK). After sensitization and amplification *in vitro*, these cells are administered to patients (16). There are two primary modalities of cell therapy: one based on genetically modified tumor antigen-specific TCRs, and the other on chimeric antigen receptors (CARs).

#### 4.1.4 Therapeutic cancer vaccines

Therapeutic cancer vaccines aim to augment the tumor-specific T cell response via active immunity (17).

#### 4.1.5 Combined targeted therapy

Combined targeted therapy has been investigated in patients who underwent second-line treatment following targeted therapies such as sorafenib. For instance, integrating immunotherapy with targeted therapy led to a tripling of response rates, a complete response rate nearing 5%, and a survival exceeding 18 months (18)). Nevertheless, the cumulative toxicity of this combination also heightened the number of severe treatment-related adverse events (TRAEs) and instances of treatment interruption due to adverse events.

#### 4.1.6 Combined local area therapy

Finally, combination with local therapies has also yielded encouraging results. For patients with advanced HCC treated with tremelimumab, the concomitant application of radiofrequency ablation for partial tumor ablation resulted in a response rate of 26%, disease stability for no less than 6 months in 45% of patients, a disease control rate of 89%, and an overall survival period exceeding 12 months (19).

### 4.2 Analysis of bibliometric results

Bibliometric research is a descriptive study that aims to analyze and visualize the complex implicit relationships among knowledge clusters, as well as to investigate the structure and evolution of scientific knowledge (20).

#### 4.2.1 Search results

##### 4.2.1.1 Implications for the current state of HCC immunotherapy

Our study elucidates the vibrant and expanding research landscape in the realm of immunotherapy for HCC, as evidenced by the extensive body of literature encompassing 4,486 papers published over the past 24 years (1999–2022). These papers, originating from 3,896 institutions across 79 countries, cumulatively received 119,523 citations, averaging 26.64 citations per paper, and achieved an h-index of 135. The expansive engagement of 23,574 authors and the dissemination of their findings across 848 journals reflect the escalating global interest and investment in this sphere.

The broad intersection of HCC immunotherapy research with other scientific domains, as suggested by the significant number of citations, highlights the extensive interdisciplinary collaboration in this field. Additionally, the surge in the number of keywords compared to previous years demonstrates the deepening and evolving understanding of HCC immunotherapy.

Our findings further reveal that emerging monitoring methodologies, pharmaceutical interventions, and treatment paradigms are being continually introduced. These advances, coupled with improved clinical outcomes, are increasingly gaining significance in HCC treatment, thereby revolutionizing the conventional therapeutic landscape.

##### 4.2.1.2 Future prospects of HCC immunotherapy

The dynamic state of HCC immunotherapy research, marked by the rapid growth in the number of publications, predicts a promising future for this field. Notably, the number of papers published in recent years has surpassed the total number of papers published during the entire decade from 2011 to 2020. This unprecedented growth rate reflects the intensified focus on immunotherapy as a viable therapeutic strategy for HCC, encouraging further exploration and innovation in this domain.

Going forward, we anticipate continued expansion and evolution in the field of HCC immunotherapy. Future research endeavors could leverage the findings from this bibliometric analysis to identify potential gaps in current knowledge and guide the direction of subsequent investigations. Moreover, as our understanding of HCC immunotherapy deepens, we foresee the development of more effective, targeted, and patient-tailored therapeutic approaches that will ultimately enhance the prognosis and quality of life for individuals diagnosed with HCC.

In conclusion, our comprehensive bibliometric analysis sheds light on the dynamic and rapidly expanding landscape of HCC immunotherapy research, and underscores the potential of this innovative treatment modality in transforming the future of HCC management.

#### 4.2.2 National analysis

##### 4.2.2.1 Distribution of hepatocellular carcinoma research output by country

Our bibliometric analysis indicates that China, Japan, the United States, Germany, Italy, the United Kingdom, and France are the leading contributors to the field of immunotherapy for hepatocellular carcinoma (HCC), in descending order of the number of published papers.

##### 4.2.2.2 China’s preeminence

China stands at the forefront, accounting for over half of the total publications. This dominant position can be attributed to several factors. Firstly, China bears a significant liver cancer burden, with the disease cases making up for approximately 55% of the global total (21). This is largely due to the high prevalence of aflatoxin toxicity and viral hepatitis infections, spurred by regional, economic, and historical-cultural contexts, such as dietary habits and past medical practices (e.g., widespread syringe exchange incident during a vaccination campaign in the 1990s). Furthermore, the Chinese government has fervently been encouraging and funding research in the field of immunotherapy, which has provided the requisite infrastructure and financial support for advancements in this area.

##### 4.2.2.3 Japan’s contribution

Japan ranks second in terms of the number of publications. Similar to China, Japan also grapples with a high hepatitis B infection rate. The government’s substantial investment in health research, coupled with distinct food preferences (like a preference for raw food), which pose health risks, contribute to the volume of HCC research output.

##### 4.2.2.4 United States’ role

The United States takes the third position. The primary etiology of HCC in the United States is believed to be alcohol consumption and obesity, fueled by the country’s dietary habits, characterized by high intake of salt, sugar, saturated fats, and cholesterol, and overeating. However, as a high-income nation, the United States has been able to make considerable investments in liver cancer treatment and research, thereby driving publication output.

##### 4.2.2.5 European countries’ participation

Germany, Italy, the United Kingdom, and France, ranked fourth, fifth, sixth, and seventh respectively, have contributed significantly to the body of research as well. The European Union aids these nations by financing various research projects and health programs aimed at promoting early HCC diagnosis, treatment, and prevention. Each country, in turn, allocates specific budgets to liver cancer-related medical services and research according to their individual circumstances.

##### 4.2.2.6 Influential factors

The variation in publication output between these countries can be attributed to several factors, including socio-economic circumstances, population size and structure, and historical events.

Socio-economic circumstances: Wealth disparity can impact the amount of investment countries can make in healthcare research. Affluent countries generally have more resources for healthcare research, and for the development of medical facilities and infrastructure. Conversely, countries with less wealth may encounter challenges such as insufficient funding, lack of medical facilities, and a shortage of skilled personnel.

Population size and structure: Countries with larger populations may require more resources to meet their healthcare needs, which could lead to a higher disease burden, diverse health challenges, and increased demand for healthcare services. The age and gender structure, population growth, and the makeup and background of immigrant populations also influence the incidence and management of liver cancer.

Historical factors: Past occurrences, including prevalence of viral infections (like hepatitis B and C), levels of alcohol abuse, conditions of healthcare and hygiene, and traditional dietary and lifestyle habits, can significantly affect the incidence of liver cancer. These historical factors have, therefore, played a substantial role in shaping the current landscape of HCC research in these countries.

#### 4.2.3 Mechanism analysis

The concentration of influential institutions (all of the top ten are located in China) underscores China’s significant contribution to the field of hepatocellular carcinoma immunotherapy. These entities are primarily prestigious universities, their affiliated hospitals, or research institutes. This indicates a potential dominance of top-tier Chinese institutions - and, by extension, globally - within this research domain. However, it is essential to recognize that a focus of research in leading institutions does not necessarily preclude participation from other entities. Smaller institutions and research centers also play pivotal roles in propelling scientific advancements in this field. Moreover, international collaboration among institutions from diverse countries and regions constitutes a considerable impetus in the forward march of hepatocellular carcinoma immunotherapy research.

#### 4.2.4 Author analysis

##### 4.2.4.1 Japanese and Chinese scholars’ contributions

Our bibliometric analysis reveals a substantial contribution from Japanese and Chinese scholars to HCC immunotherapy research. This prominence can be attributed to the high incidence of liver cancer in Asia, amplified by the cutting-edge medical technology available in these countries.

##### 4.2.4.2 Co-citation analysis of top authors

An examination of co-citation author data uncovers the critical roles of the top ten authors, each cited over 600 times, signifying their influential contributions to this field. Among these authors, Llovet JM, Finn RS, and Zhu AX emerge as pivotal figures, with 2,199, 1,444, and 1,357 co-citations, respectively.

##### 4.2.4.2.1 Llovet JM’s contributions

Llovet JM is renowned for his instrumental contributions to the development of HCC treatment guidelines and advancing our understanding of immunotherapy mechanisms (22, 23). He is notably recognized for his work on the application of sorafenib in advanced HCC (24).

##### 4.2.4.2.2 Finn RS’s research

Finn RS has made significant strides in immunotherapy research for unresectable liver cancer. His notable work includes a comparative study of Lenvatinib versus sorafenib (25) and exploration of the combined therapy of atezolizumab and bevacizumab (26).

##### 4.2.4.2.3 Zhu AX’s impact

Zhu AX has also made substantial contributions, particularly through his study of atezolizumab plus bevacizumab for unresectable HCC (26). Furthermore, he played a key role in the development of the 2018 AASLD hepatocellular carcinoma treatment guidelines (27).

##### 4.2.4.3 Evaluation of impactful research and prospective collaboration

Our comprehensive analysis, which integrates the assessment of highly cited authors, average citation frequency of papers, and literature co-citation, positions Josep M. Llovet and Richard S. Finn within the top three across all these indicators. This finding underscores their substantial contributions to HCC immunotherapy. Notably, despite Finn’s research not achieving top rank during 2010-2020 (28), his work demonstrates a prospective and groundbreaking direction in the field. Both Llovet and Finn emerge as potentially suitable scholars for future collaborative research endeavors.

#### 4.2.5 Keyword analysis

The key words were divided into four categories: adaptive immunity, immune microenvironment, immunotherapy and combination therapy.

##### 4.2.5.1 Adaptive immunity

The immune microenvironment within the liver plays a significant role in the development and progression of hepatocellular carcinoma (HCC). Key components of this microenvironment, such as NK cells, DCs, and CD8+ T cells, are crucial in orchestrating an effective immune response against HCC

##### 4.2.5.1.1 Natural Killer cells

NK cells serve a crucial function in the body’s innate immune response, acting as the primary defense against cancer. These cells possess the capability to discern between “self” and “non-self” entities, identifying aberrant cells, which leads to the eradication of transformed and malignant tumor cells. However, tumor cells have devised several strategies to elude detection and assault by NK cells. These strategies encompass upregulating ligands of NK cell suppressor receptors, generating soluble molecules, and secreting immunosuppressive factors. Such evasion mechanisms present considerable challenges to achieving successful hepatocellular carcinoma immunotherapy (14).

##### 4.2.5.1.2 Dendritic cells

DCs, as antigen-presenting cells, hold an essential role in initiating and directing adaptive immune responses. Upon antigen activation, DCs exhibit a surge in antigen uptake and mature within lymph nodes, where they facilitate the activation and polarization of naive T cells. Hence, DCs have significant potential in therapeutic cancer vaccines, demonstrating promising outcomes in recalibrating host-tumor interactions. The objective is to induce a cytotoxic antigen-specific CD8+ T cell response, which eradicates cancer cells through cellular immunity (29).

##### 4.2.5.1.3 Cytotoxic T cells

CD8+, are viewed as key effector elements in anti-liver tumor immunity. These T cells participate in mediating surveillance of precancerous hepatocytes (3). Research has demonstrated that depletion of cytotoxic T cells in mice escalates the hepatocellular carcinoma burden, underscoring their indispensable role in the immune response against hepatocellular carcinoma (30).

##### 4.2.5.2 Immune microenvironment

The tumor microenvironment plays a pivotal role in the natural progression of hepatocellular carcinoma (HCC), influencing the dynamic cross-talk between the liver’s immune system and hepatocytes. This understanding furnishes a robust theoretical foundation for therapeutic strategies that target the tumor microenvironment (31).

Both fibrosis and the immune system have significant roles in the field effects of HCC. In the context of cirrhosis, the ‘cancer field effect’ refers to the microenvironment that fosters tumor growth. Numerous genomic studies have identified major molecular elements that are upregulated within this microenvironment (3).

Hepatic stellate cells (HSCs) are integral to the liver’s response to chronic injury. Upon activation, HSCs undergo phenotypic transformations, synthesize and secrete extracellular matrix components (primarily collagen and growth factors), and contribute to endothelial cell migration, angiogenesis, and fibrosis (32). Senescent hepatocytes in precancerous lesions excrete chemokines *in vivo* that disrupt aging surveillance and weaken immune-mediated tumor suppression (33). Moreover, experimental models have underscored the importance of CD4+ lymphocytes in HCC associated with non-alcoholic fatty liver disease (NAFLD) (34), as well as the interaction between the innate immune system and the gut microbiome, both of which can expedite the development of HCC (35).

##### 4.2.5.3 Immunotherapy

Immune checkpoint inhibitors, encompassing PD-1, PD-L1, and CTLA-4, have been identified as impeding anti-tumor immune responses in solid tumors. The combination of anti-PD-1 antibodies with local area therapy or other molecularly targeted drugs has emerged as an effective treatment strategy for hepatocellular carcinoma (HCC) (36).

Over the past five years, immune checkpoint inhibitors have revolutionized hepatocellular carcinoma treatment. For instance, the combination of atezolizumab and cabozantinib has demonstrated superior progression-free survival, while tremelimumab and vatalanib have shown improved overall survival compared to sorafenib (37).

Additionally, Camrelizumab, one of the most representative drugs in negative immunomodulatory cancer therapy, was approved by the National Medical Products Administration (NMPA) in 2020 as a second-line drug for the systemic treatment of liver cancer. Camrelizumab (also known as SHR-1210) is a humanized monoclonal antibody against PD-1 that has been shown to block the binding of PD-1 to PD-L1, thereby inhibiting the immune escape of tumor cells (38).

Anti-vascular endothelial growth factor (VEGF) antibodies can normalize tumor blood vessels, thus enabling T cells to infiltrate the tumor more effectively. As a result, they serve as an optimal combination partner for immune checkpoint inhibitors (39). Immune checkpoint inhibitors (ICIs) display excellent efficacy in HCC when combined with antiangiogenic drugs, other molecularly targeted therapies, and complementary ICIs (3).

##### 4.2.5.4 Combined immunotherapy

Targeted therapy has emerged as an essential treatment option for patients with advanced unresectable liver cancer. Phase III trials have substantiated the efficacy of six systemic therapies - atezolizumab + bevacizumab, sorafenib, lenvatinib, regorafenib, cabozantinib, and ramucirumab - and these therapies have been approved for use (3).

##### 4.2.5.4.1 First-line treatment

Sorafenib, which was approved in 2007, has been the only available standard for the past 10 years. Unfortunately, sorafenib does not work as well as desired, and patients often develop resistance within six months of taking the drug (40). To address this issue, several new drugs have been developed as second-line treatments. In particular, a global open-label randomized Phase III trial (REFLECT) demonstrated the efficacy of lenvatinib, showing a longer median overall survival (13.6 months) compared to sorafenib (12.3 months) (25). Therefore, sorafenib (24) and lenvatinib (25) remain the most effective monotherapy options for first-line treatment.

The first combination regimen to improve overall survival was atezolizumab (anti-PDL1 antibody) plus bevacizumab (anti-VEGF antibody). The latest analysis revealed a median survival of 19.2 months in patients treated with this combination, as compared to 13.4 months in patients treated with sorafenib alone (26).

##### 4.2.5.4.2 Second-line treatment

For second-line treatment, regorafenib, cabozantinib, and ramucirumab are currently approved following sorafenib progression. Regorafenib, a multikinase inhibitor that targets VEGFR1-3 and other kinases, has shown improved survival (10.6 months) compared to placebo (7.8 months) (41). Cabozantinib, a multikinase inhibitor with unique activity against VEGFR2, AXL, and MET, has also demonstrated better overall survival (10.2 months) compared to placebo (8 months) (42). Ramucirumab, the only biomarker-guided therapy for hepatocellular carcinoma, has been shown to improve overall survival (8.5 months) compared to placebo (7.3 months) (43). Nivolumab, pembrolizumab, and nivolumab + ipilimumab have also been approved by the FDA for sorafenib following progression based on data from Phase Ib/II studies (3).

New combinations of various therapies have been proposed to improve the efficacy of HCC treatment. For example, a combination of PD-1/PD-L1 immunotherapy with CTLA-4 immune checkpoint inhibitors, interventional therapy TACE with targeted therapy and immunotherapy, hepatic arterial chemotherapy perfusion HAIC with targeted therapy and immunotherapy (44), and radiofrequency ablation of partial tumors combined with immunotherapy. These novel combinations have shown promising results in preclinical and clinical studies, providing new hope for patients with advanced HCC.

##### 4.2.5.5 Current impact

Adaptive immunity: Our expanding understanding of NK cells, DCs, and CD8+ T cells’ roles in HCC has informed the development of innovative therapeutic strategies. For instance, we are beginning to harness DCs’ potential in creating personalized cancer vaccines, which could reshape the HCC treatment landscape.

Immune Microenvironment: A deeper comprehension of the tumor microenvironment and the dynamic interactions between the liver’s immune system and hepatocytes have paved the way for more effective therapies. These strategies specifically target elements of this microenvironment, leading to improved patient outcomes.

Immunotherapy: Immune checkpoint inhibitors have already drastically shifted the HCC treatment paradigm. By combining these therapies with local area treatments or targeted drugs, we’ve achieved enhanced efficacy, leading to their standard application in treating HCC.

Combined immunotherapy: The successful development and approval of combination therapies - including atezolizumab + bevacizumab, sorafenib, lenvatinib, regorafenib, cabozantinib, and ramucirumab - have ushered in new treatment avenues for HCC patients.

##### 4.2.5.6 Future outlook

Adaptive immunity: The use of adaptive immune responses in immunotherapeutic approaches continues to be explored. Techniques that amplify the cytotoxic effects of NK and CD8+ T cells or utilize DCs in developing tailored cancer vaccines could significantly alter the HCC treatment field.

Immune Microenvironment: As our understanding of the tumor microenvironment deepens, we could uncover novel therapeutic targets. For example, agents that can adjust the immune microenvironment to bolster anti-tumor immune responses could offer promising treatment possibilities.

Immunotherapy: As research advances, we anticipate the development of new immune checkpoint inhibitors. Combined with other therapies, these could further enhance patient outcomes. Future work may also help identify predictive biomarkers for therapy response and resistance, leading to more effective, patient-tailored treatments.

Combined immunotherapy: Continued investigation into combination therapies, particularly those that integrate targeted therapies with immunotherapies, is expected to improve patient survival and quality of life even further.

##### 4.2.5.7 Challenges and future directions

Despite these promising advancements, several issues, such as overcoming therapy resistance and managing side effects, remain to be addressed. Moreover, enhancing our understanding of HCC’s heterogeneity is vital for identifying the best therapy or combination thereof for each patient.

Overall, the future of HCC treatment holds promise. Continued multidisciplinary research, along with the integration of precision medicine, should yield increasingly effective treatment strategies, improving patient outcomes.

#### 4.2.6 Timeline analysis of outbreak keywords

##### 4.2.6.1 Timeline shows the direction of research

Analyzing the evolution of the field of immune-related therapy, excluding highly recurring but non-directional keywords such as “*in vivo*”, “mice”, “trial”, “tumor”, and “cancer”, it is evident that:

From 1999 to 2010, the primary research focus was to elucidate the immune response of liver cancer. Research was heavily centered around understanding adaptive immunity, cytotoxic T lymphocytes, antigens, and dendritic cells.

Since 2010, the landscape of liver cancer treatment saw significant shifts. Targeted therapy emerged as a promising treatment strategy, with the approval of sorafenib in 2007 providing momentum to this field.

Post-2015, the spotlight gradually moved towards immunotherapy. Key research areas in this timeframe include 2nd line treatments (2016), PD-1 blockade (2017), checkpoint inhibitors (2018), and overall therapeutic landscape (2019).

**Figure 10:**
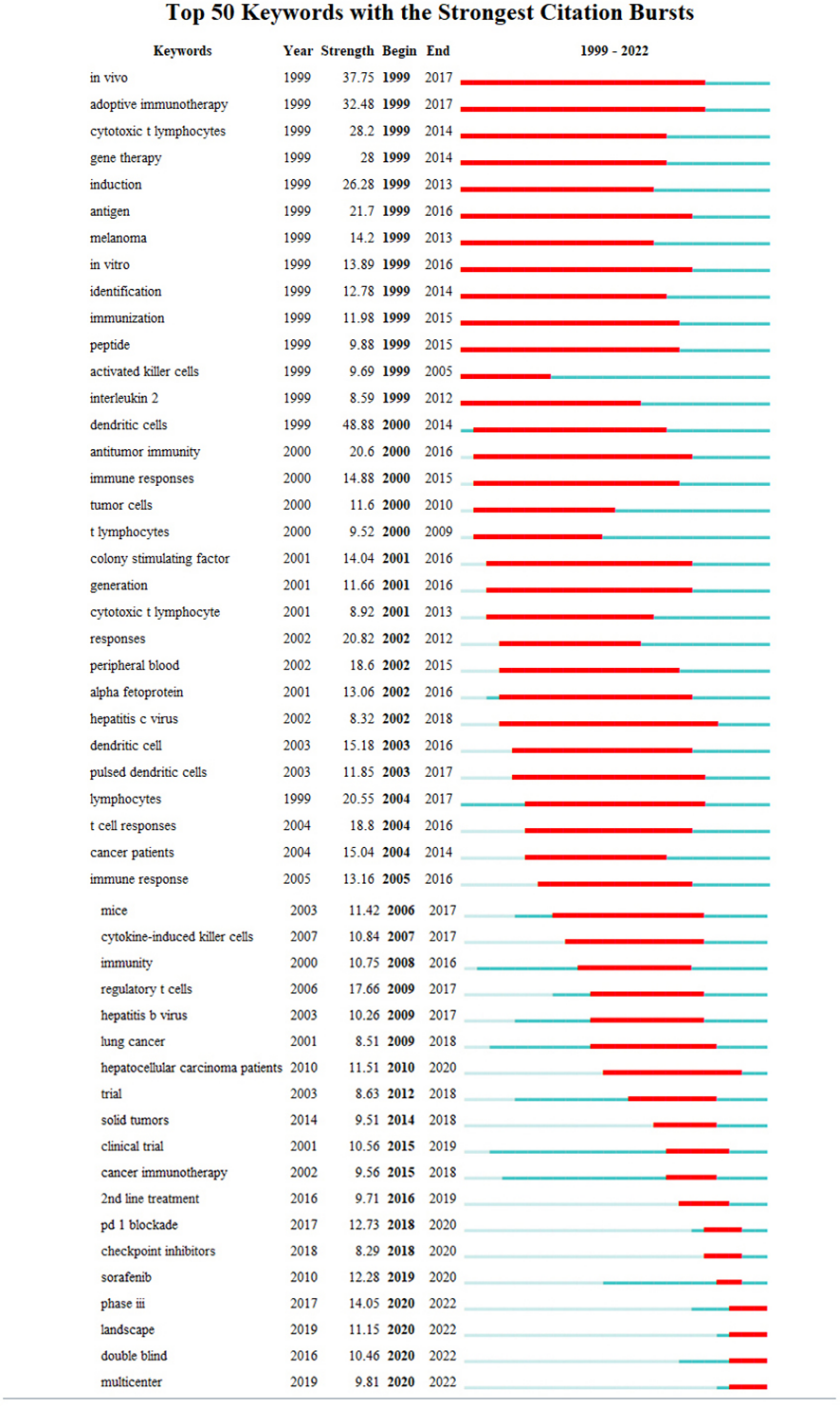
Top 50 keywords in the strongest citation burst involved in immunotherapy research in relation to HCC.

Between 2005 to 2015, research also actively investigated the role of hepatitis viruses, recognizing them as significant risk factors for hepatocellular carcinoma (**Figure 10**).

##### 4.2.6.2 Current impact

The continuous evolution of liver cancer treatment reflects the strides taken in advancing medical technology and understanding the disease’s underlying molecular mechanisms. While chemotherapy has been a staple treatment for years, the introduction of targeted therapy, epitomized by sorafenib, revolutionized the liver cancer therapy landscape. The advent of immunotherapy, especially in combination with targeted therapy, has ushered in another significant shift towards personalized medicine, bringing renewed hope to patients with advanced hepatocellular carcinoma.

Simultaneously, the identification of the hepatitis virus as a major risk factor has spurred global attention, leading to an interdisciplinary approach to liver cancer research. This collaborative method has allowed for a more comprehensive understanding of the disease’s pathogenesis, paving the way for new strategies in prevention, early diagnosis, and effective management.

##### 4.2.6.3 Future directions and expectations

As we move forward, the continuous pursuit of liver cancer research, particularly the incorporation of emerging technologies like precision medicine, gene editing, and immunogenomics, is vital in enhancing patient outcomes. The ongoing progress promises more personalized and effective patient care, opening up new horizons for liver cancer treatment.

#### 4.2.7 Literature analysis

##### 4.2.7.1 Co-citation analysis

Co-citation analysis is a bibliometric method used to identify the interconnectedness of various scientific publications within a field of study. When two or more articles simultaneously cite a previous article, a co-citation relationship is established (45). The frequency of co-citations an article receives can often be indicative of its influence within the field. High levels of co-citations suggest that a paper is frequently referred to in subsequent research, indicating its significant role in shaping knowledge and understanding within the field.

##### 4.2.7.2 Importance of highly co-cited articles

By examining the top ten co-cited articles in a particular field, we can generate an overview of the most influential ideas, research, and findings. These highly co-cited articles often form the “core” literature or the “knowledge cornerstone” of the field, as they are commonly referred to across different studies due to their groundbreaking or foundational nature. This analysis not only helps in understanding the key contributions and trends within the field but also provides insights into its evolution and direction.

##### 4.2.7.3 Analysis of liver cancer treatment literature

As demonstrated above, among the top 10 most cited literature on liver cancer treatment, 9 were clinical trials, while the remaining one was an epidemiological study.

These nine clinical studies represent important milestones in the evolution of immunotherapy for hepatocellular carcinoma, which gained momentum after Sorafenib ushered in an era of targeted therapy for liver cancer in 2007.

In 2008, Llovet’s groundbreaking discovery that Sorafenib was effective against hepatocellular carcinoma paved the way for subsequent research efforts in this area (24).

In 2013, Sangro’s study demonstrated the efficacy of Tremelimumab, an antibody that blocks CTLA-4, an inhibitory coreceptor that interferes with T cell activation and proliferation. The antibody exhibited significant antitumor and antiviral effects in patients with hepatocellular carcinoma and chronic hepatitis C virus infection (46).

In 2017, El-Khoueiry’s study revealed the potential of nabulizumab in treating advanced hepatocellular carcinoma (47). Meanwhile, Bruix’s research identified Regorafenib as the only systemic treatment option that provides survival benefits for patients with hepatocellular carcinoma whose disease has progressed during Sorafenib treatment (41).

In subsequent years, several other clinical studies have contributed to the evolving landscape of immunotherapy for hepatocellular carcinoma. In 2018, Zhu demonstrated that Pembrolizumab was effective and tolerable in patients with advanced hepatocellular carcinoma previously treated with Sorafenib (48). Kudo’s study showed that Renavatinib was no worse than Sorafenib in overall survival in patients with untreated advanced hepatocellular carcinoma (25). Abou-Alfa’s research found that Cabotinib therapy extended overall survival and progression-free survival in patients with hepatocellular carcinoma who had been treated with Sorafenib (42).

In 2020, Finn’s study provided further evidence of the effectiveness of Pembrolizumab in advanced hepatocellular carcinoma, demonstrating its continued antitumor activity and favorable risk-benefit ratio in previously treated patients (49). Additionally, Finn’s research showed that the combination of atrilizumab and bevacizumab was superior to Sorafenib alone in patients with unresectable hepatocellular carcinoma (26). These studies collectively highlight the significant progress made in the development of immunotherapy for hepatocellular carcinoma, and underscore the importance of ongoing research efforts in this field.

##### 4.2.7.4 Outlook for the future

The future of hepatocellular carcinoma (HCC) treatment looks promising, thanks to ongoing foundational research and clinical trials. The trajectory indicates further development and refinement of immunotherapies, including the discovery of new immune checkpoint inhibitors and their improved combination with other treatments. Precision medicine is expected to play an increasingly significant role, utilizing a patient’s tumor genetic and molecular profile to guide therapeutic decisions for more effective and personalized care. Continued research into the immunology and molecular biology of HCC will likely enhance our understanding of the disease, enabling the development of innovative therapies. Ultimately, this deepened knowledge will foster more personalized treatment strategies, tailoring therapy to an individual’s specific disease characteristics, thereby improving survival rates and quality of life.

### 4.3 Geographical and gender-based heterogeneity in incidence and mortality

#### 4.3.1 Global impact of liver cancer

The Global Cancer Statistics report of 2020 indeed illuminates the significant impact of liver cancer worldwide. As the sixth most common cancer and the third leading cause of cancer-related deaths, it remains a significant global health challenge, with 905,677 new cases and 830,180 deaths reported (50).

#### 4.3.2 Heterogeneity

**Figure 11:**
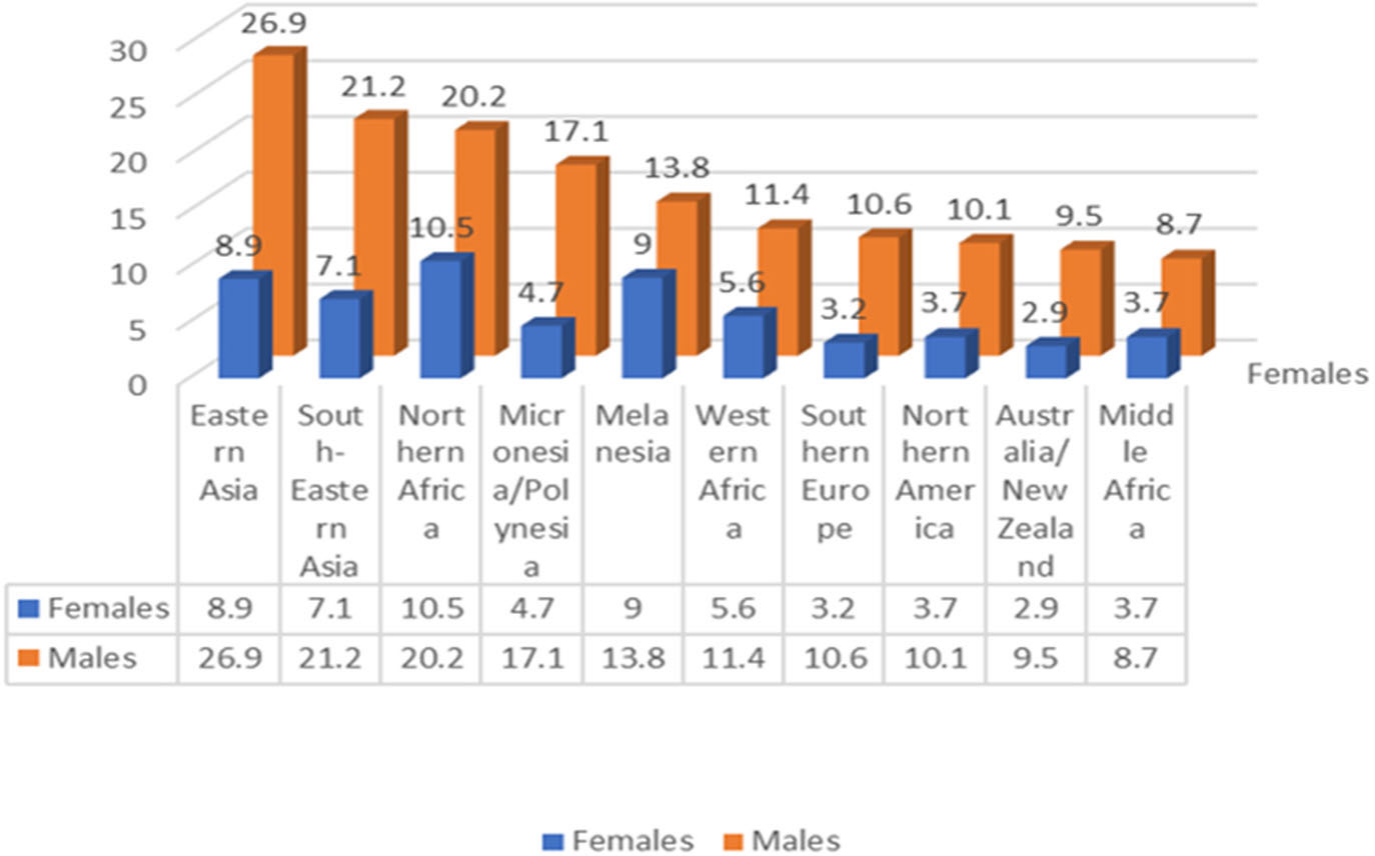
In 2020 the regional (sexage-standardized) incidence rates of liver cancer showed an additive descending order for male and female(Data from (50)).

Further examination of the data reveals notable geographic and gender heterogeneities in the incidence and mortality rates of hepatocellular carcinoma, reinforcing the importance of tailoring prevention and screening strategies to specific populations and regions [**Figure 11** (Data from (50))].

##### 4.3.2.1 Geographical heterogeneity

Geographically, hepatocellular carcinoma is especially prevalent in East and Southeast Asia as well as some areas of Africa, particularly regions with low human development indices. Chronic hepatitis B virus (HBV) infection and aflatoxin exposure represent the principal risk factors in many of these high-incidence regions, such as China and East Africa. However, in some countries like Japan and Egypt, the main risk factor is hepatitis C virus (HCV) infection.

##### 4.3.2.2 Gender heterogeneity

Gender-wise, the incidence, mortality, and cumulative risk of liver cancer are significantly higher in males, exceeding female rates by over 200%. This disparity may be attributed to biological differences and lifestyle factors between genders, though further research is needed to fully understand and address these variations.

### 4.4 Prevention and hepatitis B vaccination

#### 4.4.1 Hepatocellular carcinoma caused by chronic hepatitis B

Hepatitis B virus (HBV) remains a formidable global health challenge, with an estimated 2 billion people infected worldwide. Among this staggering number, more than 257 million individuals endure chronic HBV infection, and a distressing 30% of these patients may ultimately fall victim to the disease and its complications (51).

The progression of liver cancer in patients afflicted with HBV is orchestrated through several mechanisms, encompassing chronic hepatitis with compromised immune responses, DNA integration fostering genomic instability, disruption of cell signaling pathways by HBx protein, and HBV protein-mediated cellular oxidative stress. These multifaceted mechanisms highlight the pathogenesis of hepatocellular carcinoma and emphasize the criticality of preventing and efficaciously treating HBV infection.

##### 4.4.1.1 Chronic hepatitis with impaired immune response

Chronic Hepatitis B infection induces perpetual inflammation and immune system malfunction, precipitating prolonged inflammation, subsequent fibrosis, cirrhosis, and ultimately, hepatocellular carcinoma. Notably, within the cohort of hepatocellular carcinoma cases precipitated by HBV infection, cirrhosis is acknowledged as the predominant risk factor (52). Potential mechanisms underpinning this association might encompass hyperactivity of collagen synthesis within stellate cells and fibroblasts, in tandem with alterations in extracellular mechanisms contributing to the remodeling of the liver tumor microenvironment (36). These processes plausibly participate in the genesis and progression of hepatocellular carcinoma in patients enduring chronic Hepatitis B.

##### 4.4.1.2 DNA integration promotes genomic instability

Integration of Hepatitis B virus (HBV) into host DNA stands as a principal determinant in hepatocellular carcinoma development. Established mechanisms of HBV integration that foster genomic instability encompass (1): diminished chromosome stability subsequent to HBV integration (2), proto-oncogene insertion, and (3) mutational HBV protein expression during integration (53). These recognized processes facilitate the metamorphosis of normal hepatic cells into malignancies, underscoring the necessity of comprehending the intrinsic molecular mechanisms of HBV-related hepatocellular carcinoma.

##### 4.4.1.3 Dysregulation of HBx protein on cell signaling pathway

The HBx protein is expressed ubiquitously throughout the HBV lifecycle, acting via interactions with other proteins to yield a composite transactivation of viral DNA and cellular genes (54). The primary mechanisms of HBx action include (55) (1): integration of the hepatocellular carcinoma genome with the HBx gene, leading to diminished genetic stability (2); action on mitochondria, inducing oxidative stress (3); inactivation of tumor suppressors and activation of cell survival signaling pathways; and (4) HBx-induced epigenetic modifications contributing to tumorigenesis.

##### 4.4.1.4 Cellular oxidative stress mediated by HBV protein

Patients with chronic hepatitis B have been documented to exhibit markedly elevated levels of oxidative stress in their plasma compared to their HBV-negative counterparts, with observed levels ranging from 5.4 to 8 times higher (56). The expression of HBV-related proteins (HBsAg, HBcAg, and HBx) derived from integrated HBV DNA and cccDNA can provoke intrinsic oxidative stress in the endoplasmic reticulum and mitochondria. Additionally, the liberation of pro-inflammatory cytokines can incite extracellular oxidative stress (52).

#### 4.4.2 Hepatocellular carcinoma caused by non-chronic hepatitis B

Beyond hepatitis B, numerous other factors have been frequently linked to the development of hepatocellular carcinoma. These encompass chronic infections with hepatitis C and hepatitis D, excessive alcohol intake, consumption of aflatoxins, and obesity. These risk determinants can function independently or in synergy to amplify the likelihood of hepatocellular carcinoma development. It is paramount for individuals to be cognizant of these risk factors and take requisite measures to mitigate their risk of developing liver cancer.

##### 4.4.2.1 HCV

Hepatitis C virus (HCV) infection is known to initiate a cycle of inflammation, necrosis, and proliferation that can precipitate hepatic cell damage (57). The elements implicated in hepatocyte transformation include DNA damage mediated by reactive oxygen species, epigenetic modification, lipid peroxidation, chromosomal translocation, and mitochondrial alterations (58). Moreover, HCV escape mutants can contribute to the perpetuation of chronic infection, loss of immunomodulatory function, and promotion of HCV-mediated hepatocellular carcinoma (52).

##### 4.4.2.2 HDV

Infection with the Hepatitis Delta Virus (HDV) can precipitate the development of hepatocellular carcinoma through a multitude of mechanisms. These entail modulation of the immune response, epigenetic alterations, initiation of adaptive immune responses, modification of long non-coding RNA, and generation of reactive oxygen species (ROS). These factors can foster the development of liver cancer by inducing DNA damage, oxidative stress, and genomic instability (52).

##### 4.4.2.3 Alcohol

Despite a lack of a fully established mechanism (59), the significant role of alcohol consumption as a pathogenic factor for primary liver cancer is well-supported by a plethora of reliable experimental and clinical data (60, 61). Globally, it is estimated that 230,000 patients with alcohol-related cirrhosis have decompensated cirrhosis, while the prevalence of decompensated cirrhosis is 60,000 (62). In 2010 alone, alcohol-induced cirrhosis was responsible for 493,300 deaths, and 80,600 deaths were attributed to alcohol-induced liver cancer as a direct cause (61).

##### 4.4.2.4 Aflatoxin

One longitudinal cohort study and 13 case-control studies have documented a potent positive correlation between the consumption of aflatoxin-contaminated food and the risk of primary hepatocellular carcinoma (63). The liver is the principal target organ for aflatoxin (AF), and exposure to this toxin has been shown to elicit genetic instability, point mutations, and gene recombination during mitosis in hepatocytes. Over time, these genetic alterations can culminate in the development of hepatocellular carcinoma.

##### 4.4.2.5 Obesity

The association between obesity and cancer is extensively substantiated, with elevated incidence rates noted across various types of cancer. The ramifications of obesity are extensive, inducing changes in multiple organ systems, including endocrine and immune function alterations, thereby contributing to an escalated cancer risk. Fatty liver disease, a common sequela of obesity, is emerging as a substantial contributor to hepatocellular carcinoma in Western populations (3). As the prevalence of obesity continues its upward trajectory, so too does the incidence of fatty liver disease and its associated cancer risk.

#### 4.4.3 Clinical prevention

The incidence of HCC is strongly influenced by the prevalence of several risk factors. By understanding the mechanisms by which these risk factors contribute to liver carcinogenesis, we can develop targeted strategies for disease prevention: 1) Hepatitis B and C: Both prophylactic and therapeutic interventions, including widespread vaccination programs and antiviral treatments, have shown promising results in reducing HBV and HCV-related HCC. 2) Alcoholism: Prevention strategies focus on public health interventions to reduce alcohol consumption, including education, regulation, and counseling. 3) Obesity: Obesity-induced non-alcoholic steatohepatitis (NASH) is an emerging cause of HCC, driven by metabolic syndrome and insulin resistance. Addressing obesity through lifestyle modifications and, if necessary, pharmacological or surgical interventions, can significantly reduce HCC risk. 4) Aflatoxins: Exposure to aflatoxins, primarily through the ingestion of contaminated foodstuffs, represents a significant risk factor for HCC, particularly in regions where food storage conditions are suboptimal. Strategies to combat aflatoxin-related HCC include improving food storage methods and developing crops resistant to Aspergillus.

#### 4.4.4 Vaccination and decreased incidence of hepatocellular carcinoma

##### 4.4.3.1 Introduction of HBV vaccines and decreased infection rates

The introduction of HBV vaccines in 1982 has resulted in a significant decrease in HBV infection rates in countries that have implemented vaccination programs. As a result, the incidence of hepatocellular carcinoma, particularly in young individuals, has been notably reduced (21).

##### 4.4.3.2 HBV vaccine efficacy: insights from a randomized controlled trial

In a Chinese randomized controlled trial, the efficacy of the hepatitis B vaccine was assessed, and it was found to provide 72% (95%CI, 30-89) protection against hepatocellular carcinoma, 70% (95%CI, 23-88) protection against liver cancer-related deaths, and 64% (95%CI, 27-82) benefit in preventing liver disease-related deaths. Importantly, administering HBV vaccination at birth has shown an incredibly significant protective effect in decreasing the incidence of liver cancer (64).

##### 4.4.3.3 The vital importance of Extensive HBV vaccination campaigns

These compelling findings highlight the immense potential of HBV vaccination in combating hepatocellular carcinoma incidence and underscore the vital importance of extensive vaccination campaigns. Comprehensive vaccination initiatives not only protect individuals against initial infection, but they also substantially mitigate the risk of progression to serious complications such as hepatocellular carcinoma, particularly when administered at birth. This underscores the critical necessity of implementing universal HBV vaccination programs globally to confer optimal protection against this devastating disease.

### 4.5 Current status and future perspectives

#### 4.5.1 Evolution of hepatocellular carcinoma treatment

Indeed, the landscape of hepatocellular carcinoma (HCC) treatment has significantly evolved over the years. Traditional chemotherapy, once the mainstay of treatment, has been supplemented and even replaced by more targeted therapies, such as sorafenib, and now immunotherapy. These developments have radically transformed how HCC is managed, offering new hope for patients.

As noted, the research focus shifted considerably from the early 2000s to the present day. Initial investigations were primarily centered on understanding the immune response in liver cancer. From 2010, targeted therapy emerged as a promising alternative to conventional treatments. And more recently, since around 2015, immunotherapy has taken center stage, becoming a hot research topic in its own right.

#### 4.5.2 Potential of immunotherapy

Today, immunotherapy is recognized as an effective, promising, and innovative treatment strategy for HCC. Several immunotherapy approaches have emerged, including activated lymphocyte therapy, natural killer cell therapy, dendritic cell therapy, tumor-infiltrating lymphocyte therapy, and gene-modified T-cell therapies such as CAR T and TCR-T cell therapy. These therapies harness the body’s own immune system to recognize and fight cancer cells, offering a more personalized and potentially more effective treatment approach.

#### 4.5.3 Potential of combination therapies

Furthermore, combination therapies have shown great potential. For example, the combination of immunotherapy with targeted therapy, radiofrequency ablation, or anti-angiogenic drugs has produced encouraging results. Excitingly, novel techniques and technologies are also being explored. Examples include using nanotechnology-based electrical stimulation to galvanize the immune system to attack tumors, the development of new vaccines such as mRNA vaccines, leveraging the gut microbiome to assist in immune metabolism, metabolic reprogramming for HCC treatment, and the encapsulation and delivery of drugs using biologically engineered cell membrane-derived nanovesicles.

#### 4.5.4 Future perspectives

Looking to the future, research will likely focus on further elucidating pathological mechanisms, developing new drugs, and designing innovative combination treatment strategies. Emerging technologies, such as nanotechnology, information technology, microbiology, precision medicine, gene editing, and immunogenomics, will continue to provide new avenues for advancing liver cancer treatment. This continual evolution and advancement of HCC treatment strategies promise a more hopeful future for those afflicted with this disease.

##### 4.5.4.1 Advantage

This comprehensive study delivers a systematic analysis of the literature on immunotherapy for hepatocellular carcinoma, providing clinicians and researchers with a broad perspective on this area. To maintain objectivity, we conducted a meticulous search and included only pertinent literature. Furthermore, we employed various bibliometric tools to investigate research hotspots from diverse angles, presenting our findings in a succinct and intuitive format. Our analysis forecasts the future trajectory of the field: illuminating the mechanisms of immune response in hepatocellular carcinoma, developing additional immune checkpoint inhibitors, and exploring more options for combination therapy will likely be the focus of future research.

##### 4.5.4.2 Limitation

This study, despite offering a comprehensive overview of the literature on immunotherapy for hepatocellular carcinoma, is not without its limitations. Firstly, the literary data assembled might not be exhaustive, as we only used papers and reviews in the WoSCC with a limited set of search terms. Secondly, the low citation rate of recent high-quality literature may not fully encapsulate its academic merit due to the temporal restriction of our analysis. Thirdly, the results from breakout words, co-citation analysis, and other statistical methodologies may not necessarily represent a consensus amongst scholars in the field. Fourthly, in publications involving multiple authors, accurately assessing the contribution of individual authors proves challenging when relying on a singular, unquantified metric such as author order. Fifth, the variability of author affiliations presents considerable challenges during the bibliometric analysis process. Lastly, since this study was conducted in early 2023, it does not incorporate papers published later in 2023, though the impact of this omission on the results is likely to be minimal. Despite these constraints, this study provides a valuable macroscopic insight into the field and serves as a beneficial guide for future research.

## Author contributions

HW conceived the study and critically revised the manuscript. Z-LL conducted article retrieval, statistical analysis, data interpretation, and drafted the manuscript. YZ and BZ performed the article retrieval and data interpretation. YZ, BZ, RG, MH, Z-LL, LY wrote the manuscript. All authors contributed to the article and approved the submitted version.
