# Supplementary figures and images for: Bibliometric study of immunotherapy for hepatocellular carcinoma
Source: Front Immunol. 2023 Aug 4;14:1210802. doi: 10.3389/fimmu.2023.1210802 (PMC10436521; doi:10.3389/fimmu.2023.1210802)

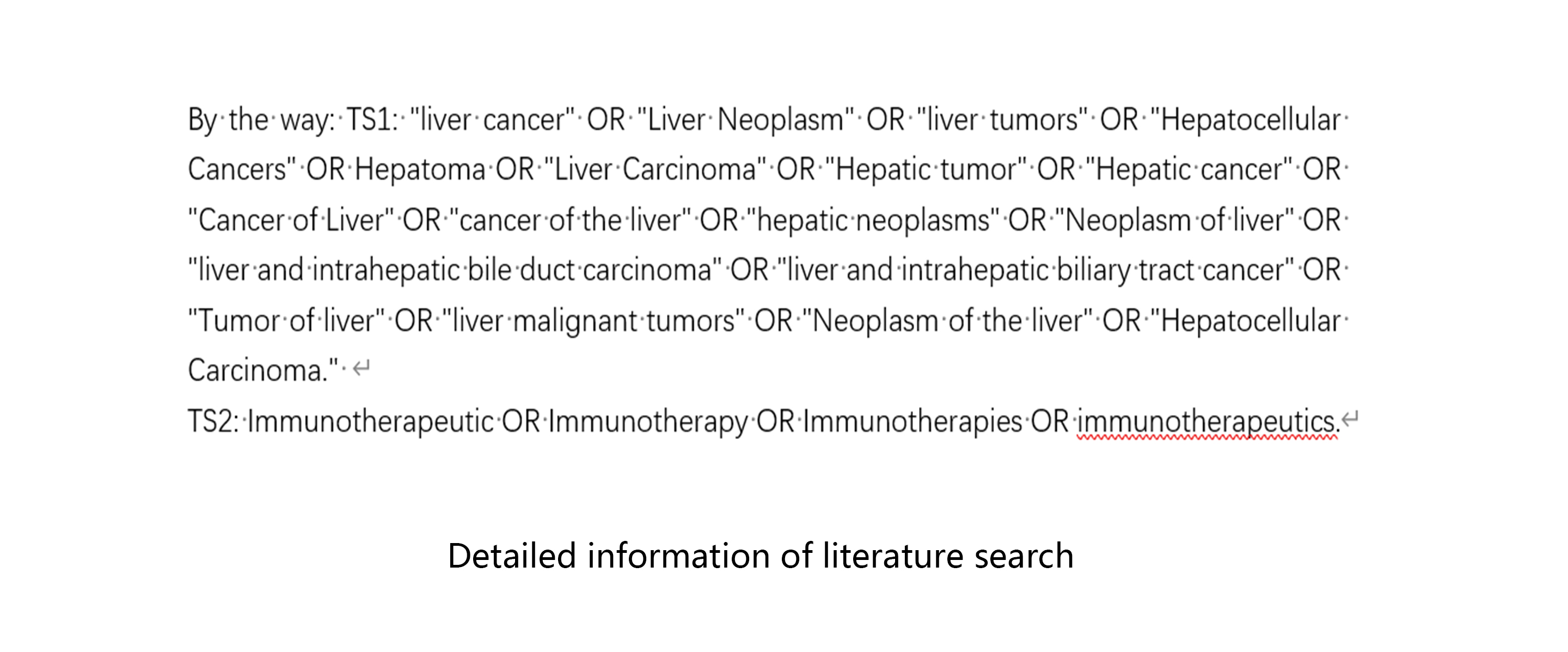

Supplement: Supplementary file 1 [file Image_1.jpeg]
